# Flexible and Transparent Ultrathin Gold Electrodes via Ion Beam Smoothing

**DOI:** 10.1002/smsc.202400272

**Published:** 2024-11-27

**Authors:** Giulio Ferrando, Carlo Mennucci, Matteo Barelli, Maria Caterina Giordano, Francesco Buatier de Mongeot

**Affiliations:** ^1^ Dipartimento di Fisica Università di Genova Via Dodecaneso 33 16146 Genova Italy

**Keywords:** biocompatible contacts, flexible electrodes, ion beam smoothing, large‐area, transparent conductive electrodes, ultrathin films, ultrasmooth films

## Abstract

Herein, a large‐area nanofabrication process is proposed for flexible, ultrathin, and ultrasmooth gold films with extraordinary electro‐optical performance, making them competitive as transparent conductive electrodes (TCEs). The approach circumvents the thermodynamic constraints associated with the physical deposition of thin film electrodes, where 3D growth and metal dewetting delay stable percolation until the deposited film thickness exceeds 8–10 nm. It is demonstrated that a postgrowth ion irradiation procedure of compact gold films with Ar^+^ beam at very low energies, around 100 eV, predominantly induces ballistic smoothing and grain boundary restructuring. This process finally leads to the formation of ultrasmooth and ultrathin gold films that remain compact even at a thickness of 4 nm, with a sheet resistance in the range of 60 Ω sq^−1^ and an optical transparency around 80%. Remarkably, the films remain percolated even at thicknesses as low as 3 nm, with a transparency exceeding 90% and a sheet resistance of 190 Ω sq^−1^. These figures are comparable to those of commercial TCEs and enable simple, scalable, all‐metal transparent contacts on both rigid and flexible substrates, with significant potential for optoelectronic applications.

## Graphical abstract

Large‐area transparent conductive electrodes based on ultrathin and ultrasmooth gold films are fabricated onto flexible substrates via ion beam irradiation. The ballistic momentum transfer is exploited by low‐energy ion irradiation to produce films as thin as 3 nm, which are endowed with high optical transparency low sheet resistance. This approach is very promising for ultrathin optoelectronic biosensing applications.

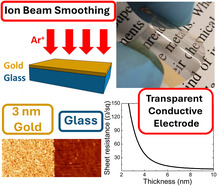

## 1 Introduction

Transparent conductive electrodes (TCEs) allow to extract and transport charge carriers while transmitting light; therefore, their development is crucial for many optoelectronics applications, e.g., electronics displays, LEDs, and photovoltaics.^[^ ^1^, ^2^, ^3^, ^4^, ^5^ ^]^ Among various materials, indium tin oxide (ITO) has been traditionally used for TCEs,^[^ ^2^, ^6^, ^7^, ^8^, ^9^ ^]^ yet it suffers from drawbacks such as high material costs, scarcity of indium, and brittleness,^[^ ^10^, ^11^ ^]^ limiting its use in the context of flexible devices or in large‐area photoconversion applications. As alternative approaches that overcome the issues of oxide‐based TCEs such as ITO, other materials such as conductive polymers, carbon nanomaterials^[^ ^12^, ^13^ ^]^ (such as graphene^[^ ^14^ ^]^), metal nanowire networks,^[^ ^15^, ^16^, ^17^ ^]^ or metal grids^[^ ^8^, ^18^, ^19^, ^20^ ^]^ can be employed.

Recently, the use of metallic films for this purpose has gained a great interest.^[^ ^21^, ^22^ ^]^ In fact, metal‐based TCEs stand out for their high electrical conductivity, potential for flexibility, stretchability,^[^ ^19^ ^]^ and the possibility to tune the electrode work function by varying the metal used. Moreover, these metallic films can be integrated into more complex configurations, such as coupling them to transparent metal oxide layers.^[^ ^23^, ^24^ ^]^ This approach can further enhance both the electrical and optical properties of the metallic film. However, the metal‐based TCEs developed so far, like metallic nanostructures and metal nanowire networks, run into issues such as high surface roughness which make them poorly suitable for thin film solar applications^[^ ^1^, ^25^ ^]^ and integration with atomically thin 2D material‐based devices. Other issues concerning metal‐based TCEs are related to the limited chemical stability and surface inertness with respect to oxidation, and to the need of an adhesion layer between the metal film and the substrate, like, e.g., in the case of Ag films.^[^ ^26^ ^]^ For these reasons, a very interesting alternative is ultrathin metallic layers based on a chemically inert metal such as gold, which is biocompatible and widely used in biosensing and bioelectronics.^[^ ^27^, ^28^, ^29^, ^30^, ^31^, ^32^, ^33^ ^]^

However, the development of large‐area and scalable techniques for the deposition of ultrathin metallic films is still an open challenge. Indeed, conventional approaches for fabricating low resistivity noble metal thin films are inadequate for producing effective TCEs. Fundamental thermodynamic constraints, related to the surface free energy and metal film dewetting, delay the onset of stable electrical percolation to a film thickness where optical transparency is already compromised. As an example, in the case of thermal deposition of gold films, stable conductance with sheet resistances around 10  Ω sq^−1^ is obtained for thickness in the range of 10 nm, for which optical transparency in the visible range is already damped to about 50%.

In this work, we show a subtractive physical approach based on low‐energy ion beam irradiation (IBI) performed in the 100 eV range on a predeposited gold film, supported onto transparent glass or polymeric substrates. In this way, thermodynamic limitations are overcome, leading to the formation of ultrathin and ultrasmooth gold films endowed with enhanced optical transparency and electrical conductivity. Unlike thermally deposited films, which show a significantly delayed stable electrical percolation due to surface dewetting, the IBI process promotes the fabrication of compact, conductive ultrathin gold films as thin as 3 nm. These films are characterized by an ultrasmooth surface with root mean square (RMS) roughness in the 1 nm range. Thanks to the very low film thickness, the optical transparency exceeds 90% in the visible range, a figure which is competitive with commercially available TCEs.^[^ ^1^, ^25^ ^]^ Remarkably, the developed scalable IBI process enables the simple growth of ultrathin gold films over large areas (in the cm^2^ order), without the use of adhesion layers.

Finally, we also show that our ion beam‐assisted approach can be performed using polymeric substrates, in view of flexible optoelectronic applications, showing competitive performance with respect to a conventional TCE such as ITO, both in terms of resistance to delamination and bending radius. In contrast to the growth conditions of oxide‐based TCEs such as ITO, the IBI process is effective at room temperature and does not require high‐temperature annealing to achieve the optimal electrical properties of layers.^[^ ^34^ ^]^ This makes the IBI process compatible for integration with thermally labile organic conductors. Moreover, thanks to the extremely low ion irradiation energy, the ion penetration depth is reduced below 1 nm, thus preserving the underlaying conductive layer^[^ ^35^ ^]^ and enabling IBI‐irradiated gold ultrathin films to be used as top contact electrode for fragile nanomaterials such as 2D semiconductors.

## 2 Results and Discussion

**Figure 1:**
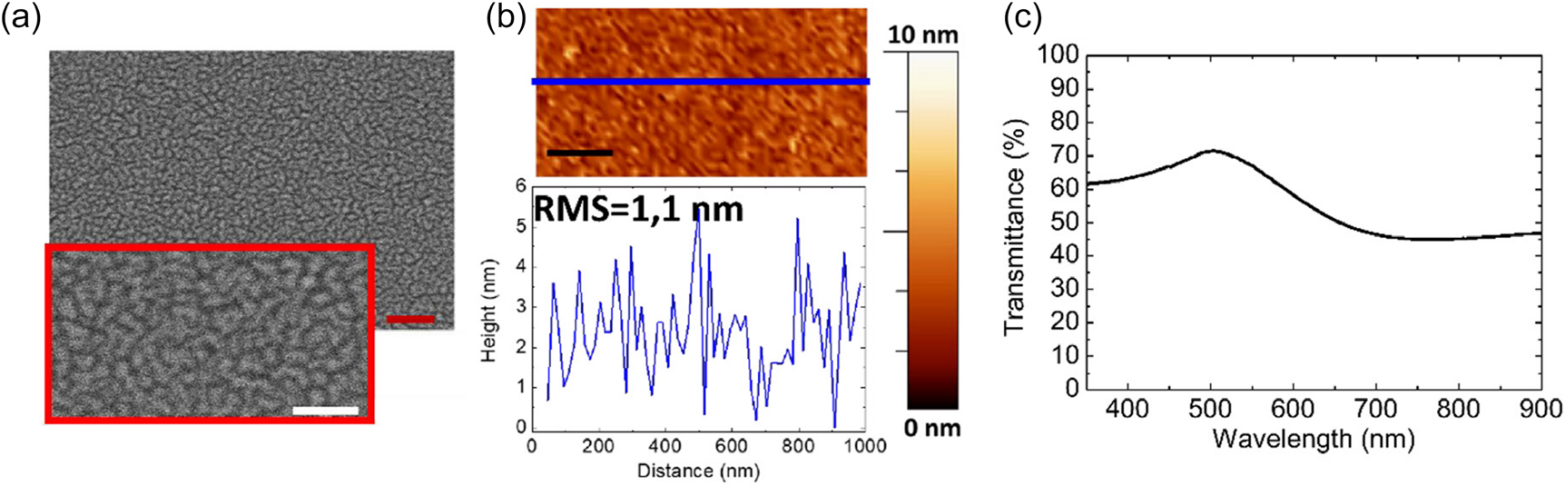
a) SEM image acquired using the backscattered electron signal of a 7 nm thick gold film. The image surrounded by a red line is a zoom of the same region. The red bar corresponds to a length of 250 nm, while the white bar in the inset to a length of 150 nm. b) AFM image (top) and selected line profile (bottom) corresponding to the blue line in the AFM image. c) Optical transmission spectra of the 7 nm tick thermally grown gold thin film.

To assess the potential of a continuous gold film as a TCE, we first analyze an as‐deposited thin film obtained through thermal deposition on a transparent soda–lime glass substrate, maintaining a constant deposition rate of 4 nm min^−1^ (more details in Experimental Section). The total deposited thickness is 7 nm, as evidenced by the analysis of a mechanical scratch shown in Figure S1, Supporting Information. For this thickness, the gold film typically forms nonpercolated islands after deposition,^[^ ^36^ ^]^ as observed in the scanning electron microscopy (SEM) image reported in **Figure** **1**a. This growth pattern is characterized by gold islands smaller than 100 nm, separated by nanogaps (dark regions in the image) wider than 1–20 nm, so that the film is electrically nonconductive. The observed morphology and the electrical characteristics of the film arise from the thermodynamic constraints imposed by the surface free energy of gold and glass. Specifically, the thermal deposition of gold follows the Volmer–Weber^[^ ^37^ ^]^ growth regime, where deposited atoms bond more strongly with each other rather than with the ones of the substrate. Under this regime, growth proceeds via the nucleation of isolated 3D islands that gradually coalesce to form a continuous and polycrystalline film. Additionally, if the film deposition is interrupted at the early stages of percolation, the high mobility of gold adatoms can lead to significant uphill mass transport due to dewetting, even at room temperature, thus forming a nonconductive film.

Despite the film of Figure 1a not showing evidence of electrical conductivity, it is indeed characterized by a very low RMS roughness of 1.1 nm, as shown in the atomic force microscope (AFM) image and its corresponding line profile in Figure 1b. From the AFM line profile, we observe that the height of the separated grains observed in the SEM image amounts to a few nanometers. The formation of disconnected gold islands is confirmed by the transmission optical spectra presented in Figure 1c, where a broad minimum at 700 nm is observed. This minimum corresponds to the excitation of localized surface plasmon resonance (LSPR) induced by the presence of the disconnected gold nanoparticles,^[^ ^38^, ^39^, ^40^ ^]^ generated by the dewetting of the gold film. It is noteworthy to emphasize that the optical transmission of the film grown by thermal deposition is poor in view of TCE applications because it remains below 60–70% across the entire visible spectral range.

Beyond thermodynamic arguments related to the Volmer–Weber balance of film/substrate surface free energy, which strongly favors metal dewetting, it is also crucial to consider the growth behavior of noble metal films like gold. These films predominantly nucleate as polycrystalline structures with basal planes in the (111) orientation. In this scenario, vertical mass transport of adatoms diffusing on the atomic terraces of the polycrystalline grains is affected by the presence of an additional energy barrier which hinders adatom descent at atomic step edges, the so‐called Ehrlich–Schwoebel (ES) effect.^[^ ^41^, ^42^ ^]^ This barrier emerges due to the different coordination number between the lower and the higher step, which hinders the downward diffusion of adatoms across step edges. Consequently, adatoms accumulate and nucleate more densely on the upper terrace, increasing the likelihood of mound formation in a so‐called kinetic growth instability.^[^ ^43^ ^]^

In this context, we explore the possibility to circumvent such thermodynamic constraints by exploiting nonthermal adatom mobility triggered by local ion collision at low energies in the IBI process. By carefully reducing ion energy to just above the sputtering threshold,^[^ ^44^ ^]^ we aim to induce hyperthermal and transient mobility of gold adatoms produced in the surface layer after ion collisions.^[^ ^45^, ^46^, ^47^, ^48^ ^]^ Specifically, the ion‐generated adatoms possess a higher energy compared to the thermal one, making them capable to more easily overcome the ES barrier which hinders downhill mass transport.^[^ ^49^, ^50^, ^51^ ^]^ Furthermore, for low‐energy ion beam irradiation at normal incidence on a mounded surface with steep local slopes, we expect to take advantage of a ballistic smoothing.^[^ ^52^ ^]^

In this process, mass transport proceeds toward the bottom of the valleys as impinging ions transfer momentum to adatoms on elevated regions, facilitating downhill movement. The final goal is to apply our IBI process to initially compact gold films to promote ion‐induced thinning and smoothing of these layers, with the aim to achieve compact and transparent films endowed with electrical conductivity in the ultrathin regime, i.e., characterized by thickness values well below 7 nm.

**Figure 2:**
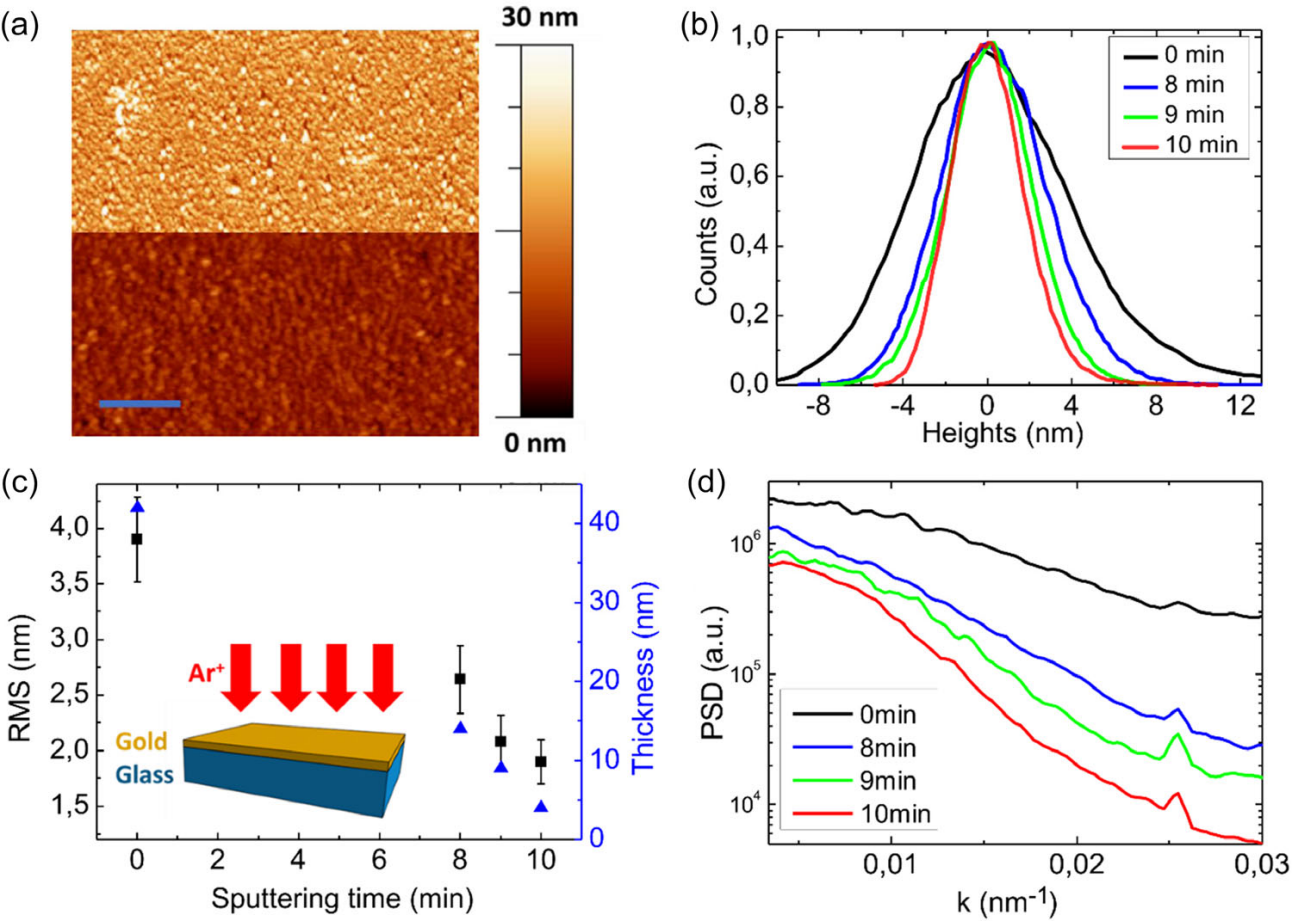
a) AFM image of an as‐deposited gold film with an initial thickness of 44 nm (top panel) and the same film after 10 min of sputtering (bottom panel). The blue line corresponds to a length of 800 nm. b) Height distribution of different AFM images acquired on the pristine film (black curve), and after different ion beam irradiation treatments corresponding to 8 min (blue curve), 9 min (green curve), and 10 min (red curve) sputtering time, respectively. c) Graph of the RMS roughness (black squares) and of the thickness of the film (blue triangles) plotted as function of the sputtering time. In the inset we show a sketch of the IBI process. d) Average of the 1D‐PSD of the AFM images corresponding to the pristine film (black curve), and to the IBI films sputtered for 8 min (blue curve), 9 min (green curve), and 10 min (red curve), respectively. The average of the 1D‐PSD is calculated along the horizontal axis corresponding to the fast AFM scan direction.

To characterize the process, we first examine the morphology of a 44 nm thick amorphous gold film subjected to a low‐energy IBI for increasing ion fluence. The initial RMS roughness of the gold film was equal to (3.9 ± 0.4) nm, where the error is calculated from the standard deviation of measurements obtained from three different AFM images acquired along the same sample to consider the variability of the surface. The film has been irradiated with a low‐energy Ar^+^ ion beam (*E* = 160 eV) at normal incidence, as depicted in the inset of **Figure** **2**c, with the ion flux maintained constant throughout the whole process at 3.2 × 10^14^ ion (cm^2^*s)^−1^. The low energy and the low flux of the beam are expected to reduce the metal thickness through the erosive action of the ion beam. In fact, the low ion energy and flux here employed induce a film erosion velocity as slow as *S* = 4 nm min^−1^ which corresponds to a sputtering yield of 1 atoms/ion. As a comparison, the erosion velocity for ion energies of 1 keV amounts to a sputtering yield of 4 atoms/ion.^[^ ^53^ ^]^

By comparing the morphology of the gold film before and after the ion treatment (top and bottom panels of Figure 2a, respectively), we observe several differences. Initially, the thermally evaporated film features some anomalous grains exceeding 30 nm in height, while the sputtered film is characterized by larger and lower grains. This behavior is consistent with the ballistic smoothing mechanism that promotes the ion‐induced mass transport in the regions with high local slopes, such as grain boundaries. As a result, small and tall grains are removed in the sputtered film. Furthermore, this morphological change is confirmed by the histogram of the height distribution in Figure 2b, which shows that distribution becomes sharper after each sputtering step, indicating a reduction in average roughness (the RMS roughness roughly scales with the half‐width of the height distribution).

This change in morphology is associated with a reduction of the RMS roughness (Figure 2c, black squares). This reduction is attributed to the decrease in the average height with sputtering time due to the predominance of downhill mass transport.^[^ ^51^ ^]^ After every irradiation step, the RMS roughness is gradually reduced from the initial value of (3.9 ± 0.4) nm to roughly half the value of (1.9 ± 0.3) nm. Each of the reported RMS roughness values is averaged between measurements performed at three different points on the sample, each 5 mm apart, to assess the uniformity of surface roughness. As an example, Figure S2, Supporting Information, presents the AFM images corresponding to the final step of sputtering. The decrease in RMS roughness goes along with a reduction in film thickness, as shown by the blue triangles in Figure 2c, measured by AFM through a scratch test (Figure S3, Supporting Information). This underlines how the low‐energy ion smoothing process enables a gradual and easily manageable thinning of the film thickness while simultaneously decreasing surface roughness. After 10 min of sputtering, the RMS roughness of the film is halved and its thickness reads 4 nm, starting from an initial thickness of 44 nm. The ion‐irradiated film is thinner than the pristine evaporated film shown in Figure 1a (thickness 7 nm); nevertheless, the film remains electrically conductive, with a resistance as low as 150 Ω measured between two Au test leads separated by 2 mm. The detected electrical conductivity is remarkable if we consider that a thermally grown gold film of the same thickness is nonconductive because its morphology is dominated by a network of nonpercolated grains.

As the analysis of the AFM images based on the RMS roughness provides only an averaged measurement of the surface roughness, in Figure 2d we also report the value of the 1D‐power spectral density (PSD) function calculated by applying a numerical fast Fourier transform (FFT) algorithm to the AFM scan line profile and averaging over all the lines. The plotted quantity (PSD of the height profile) is proportional to the square of the RMS roughness associated with different spatial wavenumbers *k = *1/*L*, where *L* is the real space wavelength of the modulations. This graph shows that the sputtering process induces a reduction of the roughness at all the lateral spatial scales plotted, and a stronger relative reduction of roughness is observed for high wavenumbers *k*, corresponding to small spatial length scale *L* in the range of 30–50 nm. These observations can be reconciled with the role of ion‐induced ballistic smoothing, which is more effective on the steeper slopes bounding small‐scale corrugations, flattening them through downhill mass transport.^[^ ^52^ ^]^ Our observations made for metal films allow to extend the more general validity of ballistic smoothing for ion irradiation in the 100 eV range which was initially demonstrated for diamond‐like carbon films.^[^ ^52^ ^]^ Under these low energy ion irradiation conditions, the erosion probability (sputtering yield) is strongly reduced, close to its threshold, while the adatom yield, i.e., the number of adatoms displaced form the bulk to the surface per incident ion, remains comparatively high and promotes surface mass redistribution.^[^ ^54^ ^]^ Additionally, under the normal ion incidence conditions here employed, the conventional morphological rippling instability, which develops at non‐normal incidence angles and leads to surface roughening, is hindered.^[^ ^45^, ^46^, ^47^, ^48^ ^]^ We rather exploit ballistic momentum redistribution following impact of single Ar^+^ ions, which results in a net downhill mass transport of gold adatoms from the top of the ridges to the bottom of the valleys.^[^ ^52^ ^]^ In this regime, the ion‐induced adatom currents reduce the height of the surface corrugations, filling the valleys between the gold grains and favoring also downhill mass transport in correspondence to grain boundaries where pinholes and gaps are formed during film growth in the percolation regime.

**Figure 3:**
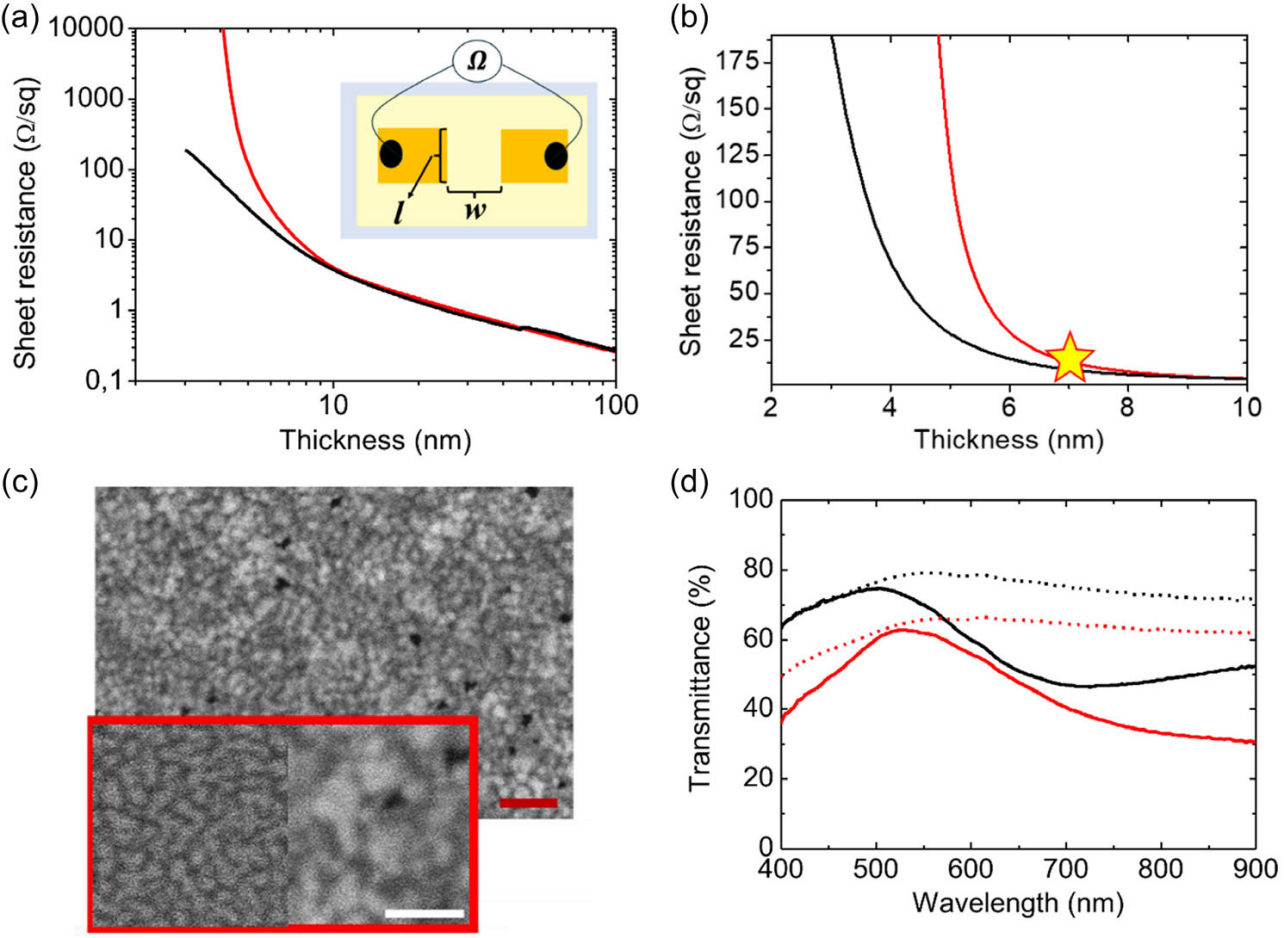
a) Evolution of the sheet resistance measured in situ during gold deposition (red line) and during the IBI process employed to achieve film thinning (black line), plotted as a function of the thickness. Data are reported in a log–log scale. In the inset we show a scheme of the in situ measurements for the gold film resistance. b) Zoom of (a) plotted in linear scale. The yellow star marks a thickness of 7 nm, where the behavior of the as‐deposited and sputtered films undergoes a significant change. c) SEM images, acquired in the backscattered electron channel, of the 7 nm thick sputtered gold film after IBI smoothing. The inset, outlined in red, provides a zoom of the same region (right part) compared to the zoom of the as‐deposited film of Figure 1a (left part). The red bar corresponds to a length of 250 nm, while the white bar to a length of 150 nm. d) Optical transmission spectra where the solid black and red lines correspond to thermally deposited gold films reading 4 and 8 nm thickness, respectively, and the dashed black and red lines corresponds to gold films threated via IBI to achieve 4 and 8 nm thickness, respectively.

The obtained film is homogeneous across the uniform sputtered area which extends about 1 cm^2^. As evidence, Figure S4, Supporting Information, shows optical transmittance measurements taken at different points spaced more than 10 mm apart from each other. As discussed later, reducing the thickness of the conductive metal film is crucial for achieving high optical transmittance, as the 7 nm thick as‐deposited film has an optical transmittance of less than 70% across the visible spectrum. However, to optimizing the IBI process, it is crucial to characterize the electrical conductivity of the film at varying thicknesses to determine the optimal threshold where the film remains conductive while maintaining good optical properties for TCE applications. The electrical properties of the films have been measured in situ by monitoring the film resistance both during the thermal deposition and the IBI thinning/smoothing process. Due to the constraints of the IBI vacuum chamber, the electrical transport measurements of the film are performed in a two terminal configuration as sketched in **Figure** **3**a, employing two lithographically deposited electrodes (yellow rectangular bars) which are 1 mm wide (*w*) and separated by a 1 mm gap (*l*). In an ideal configuration, in which the active gold film is deposited only in the square region between the electrode bars, the measured resistance would read $R=\rho \frac{l}{wd}$, where *ρ* and *d* are the resistivity and the thickness of the film, respectively. In the simple case of a square active layer (*w = l*), the above expression of *R* simplifies into $R_{\text{sq}}=\frac{\rho }{d}$ providing the sheet resistance in a straightforward manner. Our experimental configuration, however, does not allow to determine directly the sheet resistance of the active gold film because the latter is deposited over the large rectangular area of the glass substrate (light yellow rectangle in the sketch), and additionally the contact resistance ($R_{c}$) must be considered. Consequently, the resistance measured in situ can be written as $R=\beta R_{\text{sq}}+R_{c}$, where *β* is the geometrical factor that takes into account the nonsquare geometry of the film active area.

To convert the measured resistance *R* data into the sheet resistance of a square active layer, we experimentally determine ex situ after growth, the geometrical factor *β* = 0.83 by mechanical carving a square active area, as detailed in Section S5 and Figure S5a,b, Supporting Information. Furthermore, as described in Figure S5c, Supporting Information, by fitting the resistance versus thickness data according to the relation $R\left(d\right)=\beta \frac{\rho }{d}+R_{c}$, we determine the film resistivity *ρ* and the contact resistance $R_{c}$.

Following the above‐described analysis, starting from the measured resistance *R* monitored in situ and in real time, both during the thermal deposition and the IBI process, we have obtained the sheet resistance of the metallic film shown in Figure 3a,b. The final thickness for the thermal deposition experiment in Figure 3a was set to 100 nm, more than twice the value of bulk electron mean free path,^[^ ^55^ ^]^ so that the measured resistivity approaches the bulk value *ρ* = 2.35 × 10^−8^Ω m (more details in Figure S5c, Supporting Information).

After completing the deposition, the gold film undergoes controlled thinning via low‐energy IBI treatment, employing the same parameters used for the experiments of Figure 2 (beam energy 160 eV and ion flux 3.2 × 10^14^ ion (cm^2^*s)^−1^). The IBI process was stopped once the film reached a sheet resistance value of 190 Ω sq^−1^. The ultimate thickness was determined through ex situ AFM examination of a mechanical scratch on the sample, as reported in Figure S6, Supporting Information. By assuming a constant sputtering yield for the gold film, we can plot the sheet resistance as a function of film thickness, as shown in Figure 3a.

In Figure 3a,b we have evidence of a significant hysteresis in the measured sheet resistance when proceeding in sequence, first through deposition (thickness increase) and subsequently through IBI (thickness decrease). We stress that in this real‐time dynamic measurement, the electrical percolation appears at lower thickness values in comparison with a static measurement performed on a film of the same thickness after the gold deposition flux is interrupted. This occurs because the gold film is in a metastable morphological configuration during the dynamic deposition. Specifically, if the thermal growth is interrupted for thickness below 7 nm, such as in Figure 1a, the film promptly dewets and becomes nonconductive.^[^ ^38^, ^39^ ^]^ In our case, the deposited film (red line) exhibits a conductive behavior only for thicknesses exceeding 5 nm, while the IBI thinned film (black line) exhibits stable conductivity below this threshold, achieving a resistance below 200 Ω sq^−1^ at a thickness of 3 nm.

Notably, as illustrated in Figure 3b, a significant splitting of the resistance curves is observed for thickness values below 7 nm. To further explore this difference in the electrical behavior, we performed an SEM analysis of the surface of a IBI film whose thickness reads 7 nm (Figure 3c), corresponding to the yellow star reported in Figure 3b. Figure 3c shows a top‐view SEM image of the sputtered film, which differs strongly from the corresponding as‐deposited film counterpart shown in Figure 1a. The inset of Figure 3c offers a direct comparison between the two films. The ion‐irradiated film (image on the right) is characterized by larger grains whose size is in the range of 50–100 nm, while the thermally deposited film (image on the left) is characterized by gold islands, which are typical of a dewetted film and inhibit electrical conductivity. Overall, the IBI‐processed film shows grains with interconnected boundaries, and just a couple of disconnected pinholes reaching the substrate are visible. Correspondingly for the film treated with the IBI process, as thin as 3 nm, the sheet resistance is still low in the range of 200 Ω sq^−1^. It is thus possible to conclude that the low energy IBI process allows the overcome the thermodynamic and kinetic constraints which drive morphological evolution of thin film in the conventional thermal growth process.

We characterized the ultrathin gold film fabrication process also in terms of optical properties across the UV–vis–NIR spectral range. In Figure 3d, we show the optical transmittance spectra of IBI‐treated and as‐deposited films for two different estimated thicknesses of 4 nm (black lines) and 8 nm (red lines). Concerning the as‐deposited films (solid lines), a transmittance minimum is visible at 700 nm for the thinner film (black solid line), which becomes broader and shifts to higher wavelengths for the thicker film (red solid line). This optical feature is due to the excitation of a LSPR supported by the gold islands in the nucleation regime. The observed redshift of the LSPR resonance as the thickness of the deposited film increases is due to the coarsening process of the gold islands.^[^ ^56^ ^]^ A comparison between the ion irradiated (dashed lines) and the as‐deposited films (solid lines) reveals enhanced optical transparency in the case of IBI‐treated films across a broadband visible and NIR spectrum because they do not support plasmonic resonances.

**Figure 4:**
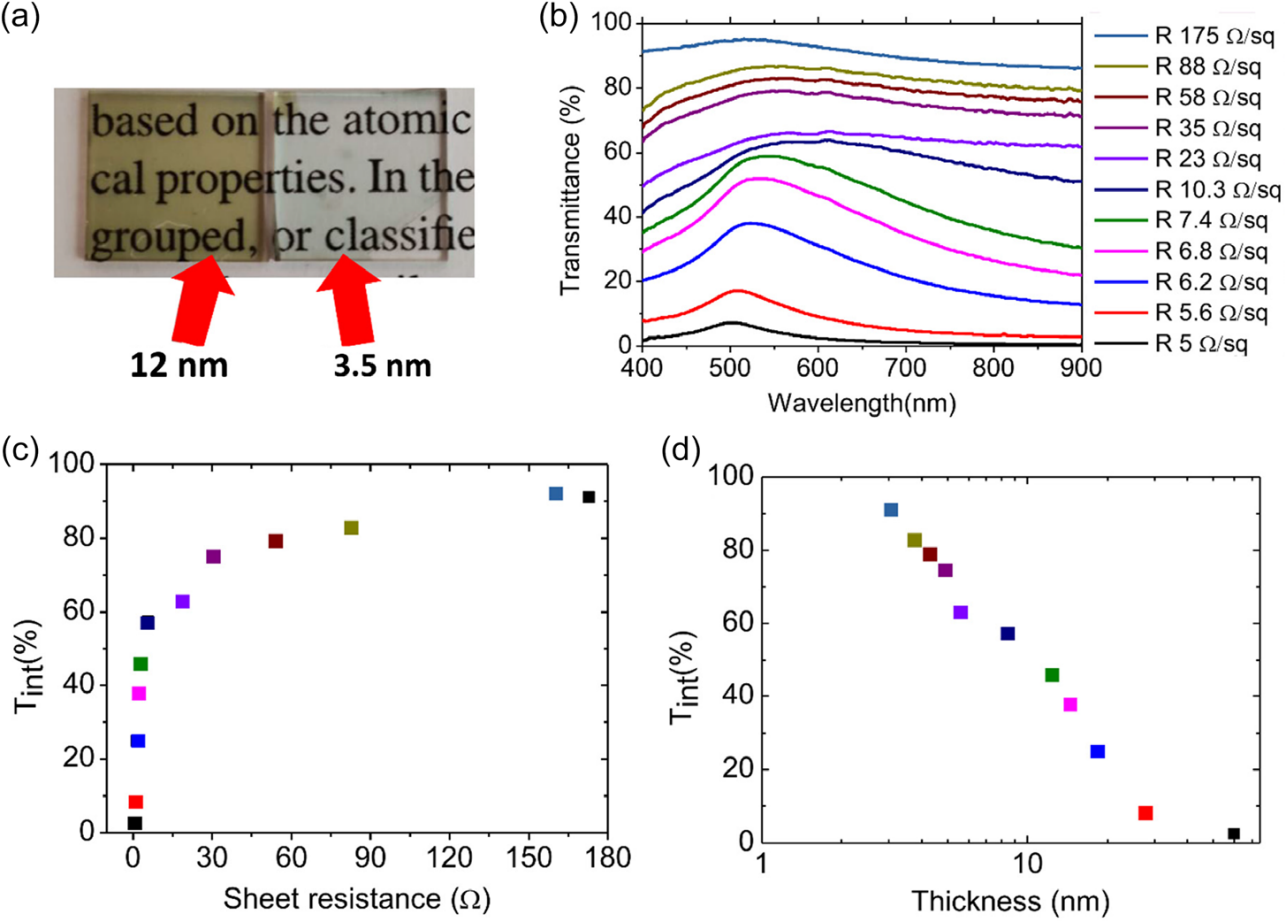
a) Picture of two gold film reading 12 and 3.5 nm thickness, respectively. b) Transmittance spectra of the film detected in situ during the IBI process, in correspondence of different sheet resistance values of the film, as shown in the key. c) Integrated optical transmittance of the film in the visible and NIR spectrum (from 400 to 800 nm wavelength) plotted as function of the sheet resistance of the film. d) Integrated visible and NIR optical transmittance for different values of the film thickness. In (c,d) the color of the squares matches the color of the spectra corresponding to the points shown in (b).

The thinner (4 nm) sputtered film (black dashed line) is characterized by a transmittance value that exceeds 80% at a wavelength of 525 nm, while in the wavelength range between 400 and 800 nm it shows an averaged optical transmittance of about 75%. The 4 nm IBI processed transparent film is also highly conductive showing a sheet resistance of about 60 Ω sq^−1^, a remarkable figure in view of TCEs applications. Nonetheless, it is evident that the film thickness plays a crucial role in determining optical transmittance, particularly in the ultrathin regime. This dependency is highlighted when comparing the distinct characteristics of two films shown in **Figure** **4**a. The image clearly showcases the enhanced optical transmittance achieved through the IBI smoothing process while also highlighting the uniformity attained across areas spanning ≈1 cm^2^.

To further optimize the optical behavior of the sputtered gold films, we detected the optical transmittance of the film in situ during the IBI process. More in detail, at fixed values of resistance, reached after a certain sputtering time, we acquired the corresponding in situ optical spectrum. The thickness of each sample was estimated off the sheet resistance behavior depicted in Figure 3a. All spectra were normalized with respect to the transmittance of the bare glass substrate, enabling the assignment of resistance values to the different optical spectra, as shown in Figure 4b.

From the analysis of the acquired in situ spectra, it is possible to observe that for high resistance values (corresponding to a low film thickness) no optical features can be attributed to a LSPR.

This confirms once more that for very low thicknesses the sputtered film is still compact. Furthermore, examination of the transmittance spectra reveals that for a film with a sheet resistance of 5 Ω sq^−1^, the visible transmission is damped below 10%.

However, as the resistance increases above 50 Ω sq^−1^, the transmission strongly increases, exceeding 80%. Finally, with an increase of the resistance to 190 Ω sq^−1^, the optical transmission increases above 90%.

Figure 4c displays the integrated transmission across the visible and NIR spectrum, ranging from 400 nm to 800 nm wavelength, plotted as a function of the measured sheet resistance to highlight the evolution of the optoelectronic properties of the film during the IBI process. The integrated transmission (*T*_int_) between the wavelengths $\lambda_{1},\lambda_{2}$ is defined as

(1) $$T_{\text{int}}=\frac{1}{\lambda_{2}-\lambda_{1}}\sum_{\lambda =\lambda_{1}}^{\lambda_{2}}T\left(\lambda \right)$$

Therefore, this is a parameter that encapsulates the film's transmission across the whole visible spectral range. It is possible to see that the *T*_int_ strongly changes for a sheet resistance below 30 Ω sq^−1^, while the increase is less pronounced above this threshold. In proximity of this threshold, a compromise between optical transmission and sheet resistance can be thus achieved. Specifically, it becomes feasible to prepare a film endowed with *T*_int_ ≈ 80% and a sheet resistance of 30 Ω sq^−1^.

Additionally, by exploiting the measured sheet resistance values for different thicknesses as depicted in Figure 3b, we can estimate the *T*_int_ values corresponding to different film thickness, as shown in Figure 4d. This graph highlights the versatility of the IBI technique, which allows for the precise control of both the sheet resistance and the optical transmission by varying the thickness of the gold film.

We have so far demonstrated the versatility of the low energy IBI treatment in achieving conductive ultrathin and ultrasmooth metal films with finely tunable sheet resistance and optical transmittance. In addition, we also stress that metallic nanoelectrodes are robust against bending of the supporting substrate due to the metal ductility, making them an ideal platform for flexible TCEs fabrication.

**Figure 5:**
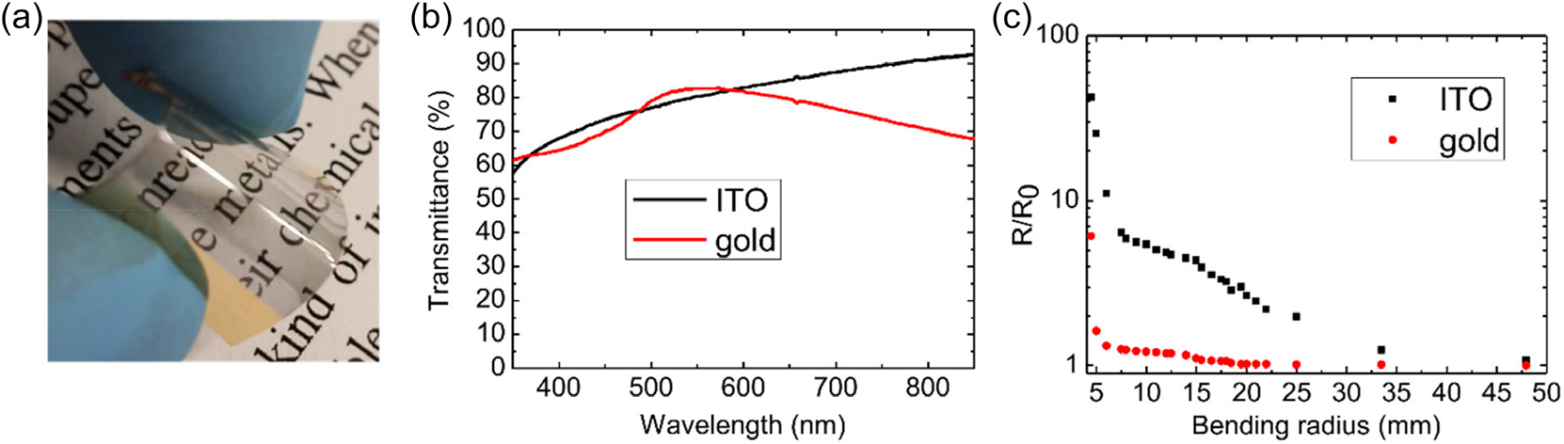
a) Picture of the flexible gold electrode. b) Comparison between the optical transmission spectrum of a flexible ITO electrode (black line) and that of a flexible gold electrode (red line). c) Comparison of the resistance (*R*) of the film normalized to its initial resistance (*R*_0_) for different bending radius. The red dots are relative to the gold film and the black dots are relative to the ITO film.

To prove this point, as a proof of concept, we compare a flexible electrode based on an IBI smoothed gold film with a flexible ITO film. Both films are deposited onto a polycarbonate substrate. For the electrode based on sputtered gold, the IBI treatment is stopped when a sheet resistance close to 100 Ω sq^−1^ is achieved. In this way, it is possible to obtain a flexible electrode that combines a good sheet resistance with a good optical transmission in the visible spectral range, as can be seen in **Figure** **5**a.

To test the bending resistance of the gold electrode, its performances were compared with those of the ITO film. The latter is deposited via RF sputtering technique (more details in Experimental Section) resulting in a film thickness of 50 nm. It shows electrical properties that are comparable to those of the gold film, with a sheet resistance of 140 Ω sq^−1^. Furthermore, the optical transmittance of the two electrodes is comparable in the visible spectral region, between 400 nm and 600 nm wavelength, as reported in Figure 5b.

In Figure 5c, we show the electrical test of the conductive films modified by bending stress. In particular, the ratio between the initial resistance of the film (*R*_0_) and the resistance of the film during the bending process (*R*) is plotted as a function of bending radius. The graph shows a strong difference between the ITO and the gold film, that is evident from the first stages when the bending radius decreases from 35 to 15 mm. The resistance of the ITO film increases fivefold compared to its initial value, while the resistance of IBI processed gold remains unchanged. This difference becomes stronger when the bending radius is decreased down to 6 mm because the ITO film resistance increases up to 10 times with respect to its initial value, while the resistance of the IBI processed gold film is almost stable, with a slight increase by a factor 1.2.

To confirm this result, we also analyzed the resistance of the IBI‐processed gold film after 1200 bending cycles with two different bending radii (4 mm and 6 mm), as shown in Figure S7, Supporting Information. Additionally, we compared the performance of the ultrathin gold film with an ITO film subjected to a 6 mm bending radius for the same bending cycles amount. For both bending radii tested, the IBI‐treated gold film exhibited superior resistance during bending. Specifically, the gold film for a 4 mm bending radius showed a resistance that was 6 times better than that of the ITO film, while for 6 mm bending radius, it was 20 times better. This comparison highlights the potential of IBI‐irradiated ultrathin metal films as TCEs in flexible ultrathin optoelectronic devices.

## 3 Conclusion

In this work, we present a physical method for producing ultrathin and ultrasmooth gold films that are competitive as flexible TCEs. These continuous films are biocompatible and stable under ambient conditions, making them particularly valuable for applications beyond optoelectronics, such as in biology and neuroscience. For this purpose, the growth of conductive ultrathin gold films, thinner than 5 nm, is crucial to achieve high optical transmittance over a broadband visible and NIR spectrum. To overcome the thermal dewetting effect that occurs for thin films grown by conventional physical deposition techniques, we develop a novel large‐area ion beam smoothing and thinning treatment based on low energy and a low flux IBI. This technique enables the fabrication of conductive, homogeneous, large‐area, ultrathin, and ultrasmooth gold films and can be potentially scaled up to the industrial level for real world technological applications.

A key advantage of the IBI treatment is that it does not involve high‐temperature annealing processes to reach optimal electrical properties, unlike oxide‐based TCEs such as ITO layers, making it compatible with thermally sensible organic conductors. Additionally, for low‐energy Ar^+^ ion beams (in the 100 eV range), the ions penetration depth in the conductive layer is limited to below 1 nm, thus allowing the formation of ultrathin metal top contact electrodes without degrading the underlying materials, a critical issue in view of the integration of the contacts with labile materials such as 2D semiconductors.

In our work, we demonstrate the capability to reduce the thickness and the RMS roughness of a gold film by using the IBI treatment, achieving roughness values as low as ≈1 nm. By means of optoelectronic in situ characterization, we compare the response of a IBI‐treated film with that of a thermally deposited one. The results reveal that this technique maintains film compactness and conductivity even at thicknesses well below 7 nm, where conventional gold films dewet at room temperature. In this ultrathin regime, the smoothed film optimally combines electrical conductivity and optical transparency, with a maximum transmittance exceeding 90% in the visible spectrum, rivaling commercial ITO films.

We finally present as a proof‐of‐concept a metal‐based flexible electrode that shows improved resistance to bending stress compared to an ITO electrode grown onto an identical polycarbonate substrate. The gold film retains conductivity even when subjected to bending radii over 7 times higher than those sustainable by the ITO film. These findings underline the versatility of our proposed technique, facilitating the fabrication of flexible TCEs from compact metals.

We highlight the potential extension of this technique to the employ of various metals (e.g., Ag, Al, Cu) with different work functions, which are promising candidates for diverse technological applications. These ultrathin electrodes can play a crucial role in advanced optoelectronic devices based on 2D materials, which demand ultrasmooth contact surfaces, along with a tunable work function that can be attained by properly choosing the electrode material.

This large‐area approach for metal‐based ultrasmooth thin‐film electrodes can thus have impact in a wide range of applications ranging from flexible optoelectronics to photodetection and biosensing.

## 4 Experimental Section

4.1

4.1.1

##### Deposition Procedure

Gold (purity 99.99%) has been thermally evaporated from a heated alumina coated crucible. The deposition has been performed at room temperature within a custom designed vacuum system with a base pressure in the low 10^−6^ mbar range. The gold films were grown on a soda–lime glass (Thermo Scientific) and the thickness deposited was monitored by means of a calibrated quartz microbalance.

##### Sputtering Procedure

The polycrystalline gold films were sputtered with a defocused Ar^+^ (5 N purity) ion beam using a gridded multiaperture source (Tectra Instruments). Surfaces were irradiated at a constant flux of 3.2 × 10^14^ ions cm^−2^*s (measured in a plane orthogonal to the beam direction) and at an ion energy of 160 eV. Samples were exposed to the ion beam at a normal incidence angle. A biased tungsten filament was used to counteract surface charging by emitting electrons through thermionic emission.

##### Sheet Resistance Measurements

Resistance measurements were performed in real time during the sputtering process by means of a digital multimeter (HP 3478A) coupled to a Lab‐view data acquisition tool.

##### Optical Transmittance Measurements

Optical transmittance spectra have been quantitatively investigated by means of a solid‐state spectrometer (HR4000, Ocean Optics) operating in a wavelength range comprised between 300 and 1100 nm.

##### Morphological Analysis

The surface topography was investigated ex situ by atomic force microscopy (Nanosurf Mobile S) operated in tapping mode. Using the WSxM software^[^ ^57^ ^]^ we calculated the RMS roughness and of the 1D‐PSD function, calculated by applying a numerical FFT. To achieve a direct evaluation of the thickness of the residual metal film, we have investigated by AFM portions of surfaces where the film was mechanically scratched using a micrometric tip.

##### ITO Deposition

ITO layers have been grown by a custom‐made RF sputtering system using a 2″ ITO target. The RF sputtering experiment is performed in argon atmosphere at a power *P* = 10 W, sample‐target distance *d* = 8.5 cm, and an initial pressure of low 10^−6^ mbar. The thickness of the sample is monitored during the sputtering process by means of a calibrated quartz microbalance.

##### Statistical Methods

The statistical parameters which describe the surface topography (RMS roughness, 1D‐PSD) were derived using the WSxM software^[^ ^57^ ^]^ by averaging at least three AFM images per region. The error bars represent the standard deviation. The fitting of the resistance versus thickness data (Figure S5c, Supporting Information) was performed using a nonlinear least squares fitting routine implemented in the Origin software package.

## Conflict of Interest

The authors declare no conflict of interest.

## Author Contributions

**Giulio Ferrando**: Data curation (lead); Formal analysis (lead); Investigation (lead); Writing—original draft (lead). **Carlo Mennucci**: Investigation (equal); Methodology (equal); Writing—review and editing (equal). **Matteo Barelli**: Funding acquisition (supporting); Methodology (equal); Supervision (supporting); Writing—review and editing (equal). **Maria Caterina Giordano**: Funding acquisition (equal); Methodology (equal); Resources (equal); Supervision (equal); Writing—review and editing (equal). **Francesco Buatier de Mongeot**: Conceptualization (lead); Funding acquisition (lead); Supervision (lead); Writing—review and editing (equal).

## Supporting information

Supplementary Material

## Data Availability

The data that support the findings of this study are available from the corresponding author upon reasonable request.
