# Supplementary material for: Flexible and Transparent Ultrathin Gold Electrodes via Ion Beam Smoothing
Source: Small Sci. 2024 Nov 27;5(1):2400272. doi: 10.1002/smsc.202400272 (PMC11935187; doi:10.1002/smsc.202400272)
Supplement: Supplementary file 1 — Supplementary Material [file SMSC-5-2400272-s001.pdf]

Supporting Information

Flexible and transparent ultrathin gold electrodes via ion beam smoothing

Giulio Ferrando, Carlo Mennucci, Matteo Barelli, Maria Caterina Giordano, Francesco Buatier de Mongeot

1. Thickness calibration of an as-deposited gold film

Mechanical scratch of the as-deposited gold film, analyzed by means of WsXM software (**Figure S1a**). The thickness of the film is determined by the histogram of the heights (**Figure S1b**).

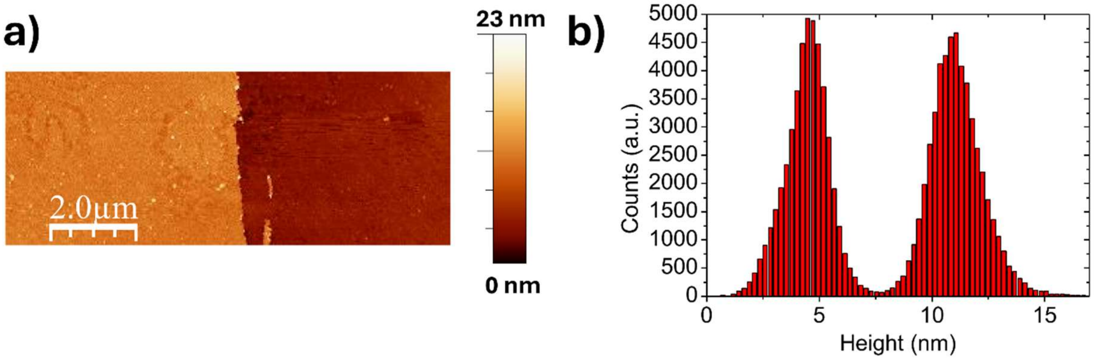

Figure S1. a) mechanical scratch of an as-deposited gold film b) Histogram of the heights relative to the AFM image reported in panel a.

2. Assessment of the RMS roughness after the IBI treatment

Figure S2 shows the AFM images acquired after the final sputtering step depicted in **Figure 2c**. The RMS values measured at different points are 1.796 nm, 1.719 nm, and 2.259 nm, respectively. The average RMS value calculated from these measurements is 1.9 ± 0.3 nm.

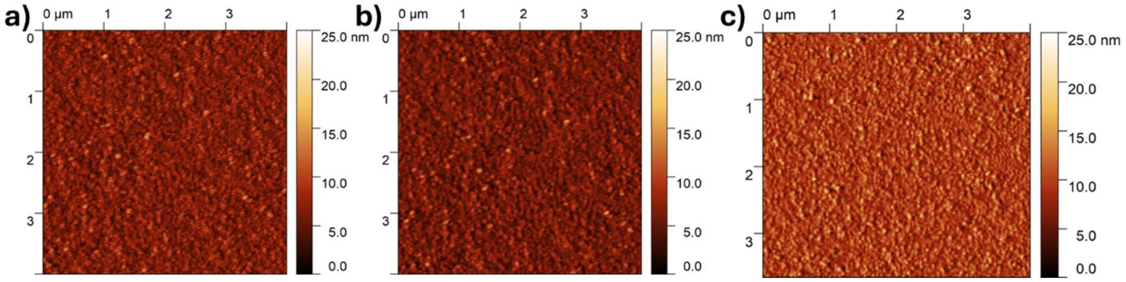

Figure S2. a,b,c) AFM images relative to the last sputtering step reported in Figure 2c.

3. Thickness measurement after the IBI treatment of Figure 2c

In **Figure S3** is reported the thickness calibration of a gold film with an initial thickness of 44 nm (**Figure S3a,b)** as well as the measurements taken after the IBI treatment. These measurements are derived from AFM images of the mechanical scratches and their corresponding height histograms. Specifically, data acquired after 8 minutes (**Figure S3c,d**), 9 minutes (**Figure S3e,f**), and 10 minutes (**Figure S3g,h)** of IBI treatment are shown.

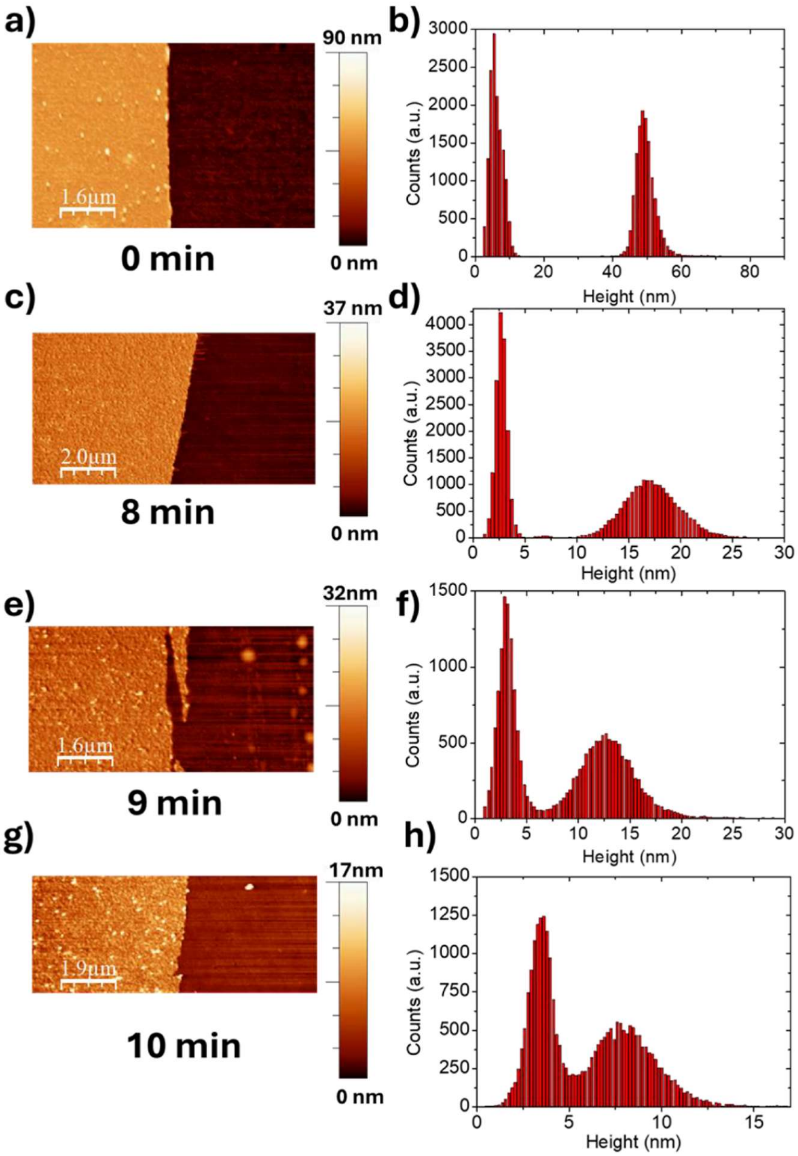

Figure S3. a,c,e,g) Mechanical scratch of the sputtered gold film respectively after 0, 8, 9, 10 minutes of IBI treatment. b,d,f,h) Histogram of the heights relative to the AFM image (reported in panel a,c,e,g).

4. Homogeneity of the optical transmittance after the IBI treatment

The homogeneity of a smoothed film with a thickness of 10 nm has been verified by measuring optical transmittance spectra in different positions, separated by more than 6 mm (as reported in **Figure S4a**). Form **Figure S4b** it is possible to observe that the optical spectra change only negligibly across the sample in areas of several cm^2.

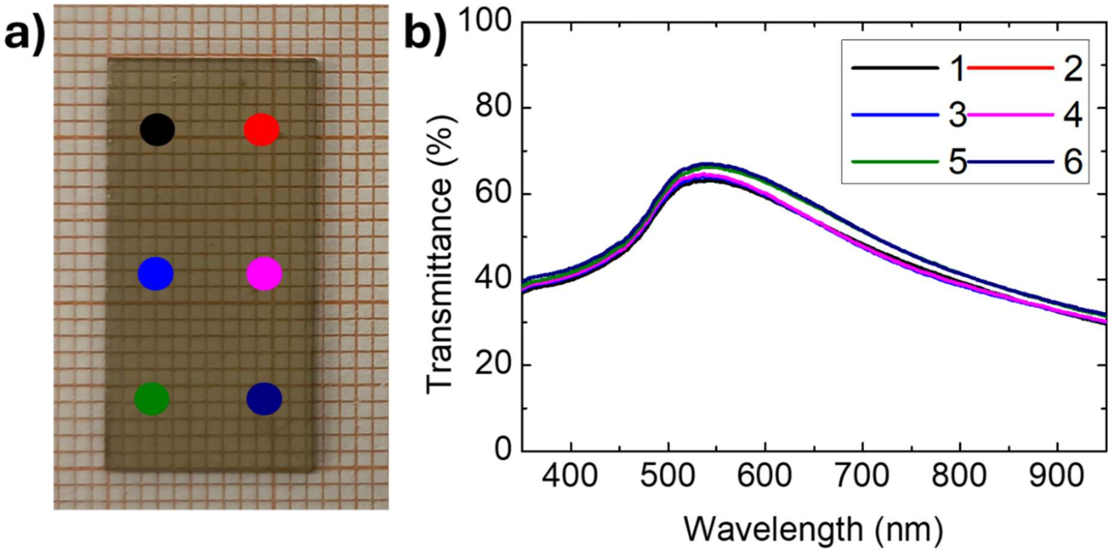

Figure S4. a) Photo of a gold film 10 nm thick. b) Different spectra corresponding to the dots reported in panel a.

5. In-situ sheet resistance measurement - determination of the geometrical factor β

The resistance measured with a 2-probe configuration (R), reported in the sketch of **Figure 3a**, can be linked to the sheet resistance (R_{sq}) of the film by the following relationship:

$$R = \beta R_{sq} + R_c$$

Where β is a geometrical factor and R_c is the resistance of the contacts. Specifically, β takes into account that the active gold film deposited during the in-situ measurement does not have an ideal square geometry (light yellow rectangle in sketch 1 of **Figure S5a**).

To obtain the value of β, first we perform the ex-situ measurement of the resistance R_1=171 Ω of an ultra-thin gold film deposited on the whole substrate with a non-square geometry (sketch 1 in **Figure S5a**). Afterwards we performed a mechanical scratch of the gold active film to reach the square configuration (sketch 2 in **Figure S5a**). During this process, we measured the resistance (**Figure S5b**), recording a final value of R_2 = 206 Ω. Since the contact resistance (R_c ≈ 1 Ω) is much lower than R_1 and R_2, β can be determined as the ratio of R_1 to R_2, yielding a value of 0.83.

After correcting the measured resistance with the geometrical factor β we determined the contact resistance R_c of the electrodes during the in-situ measurement by fitting the experimental measured resistance with the function $R = \frac{\beta\rho}{d} + R_c$, where ρ is the resistivity of the gold film and d is the thickness of the film. For the fitting, we used resistance values measured in the thick film regime (between 70 nm and 100 nm), where the film's resistivity approaches the bulk value. The result of the fit is presented in **Figure S5c**, and corresponds to a contact resistance R_c=2.5 Ω, and a value of ρ =2.48*10^{-8} Ω m, that is very close to the nominal bulk resistivity value of 2.35*10^{-8} Ω m.

To independently verify the accuracy of our geometrical correction factor β, we measured the sheet resistance of a reference gold film with a commercial 4-point probe system (Ossila P2010A2), obtaining R_{sq} =4.60±0.09 Ω\sq. We then compare this value with a measurement performed ex-situ using the same contact pads employed for 2-probe in-situ measurements, but adding two more voltage sense leads in a 4-probe configuration (as reported in the sketch of **Figure S5b**) which eliminates the contact resistance. Considering the geometrical factor β=0.83 we obtain R_{sq} = 4.24 Ω\sq. The observed 8% difference between the two sheet resistance values represents the experimental accuracy of the geometrical β factor evaluation, which is mainly determined by geometrical inaccuracies in the mechanical scratching process.

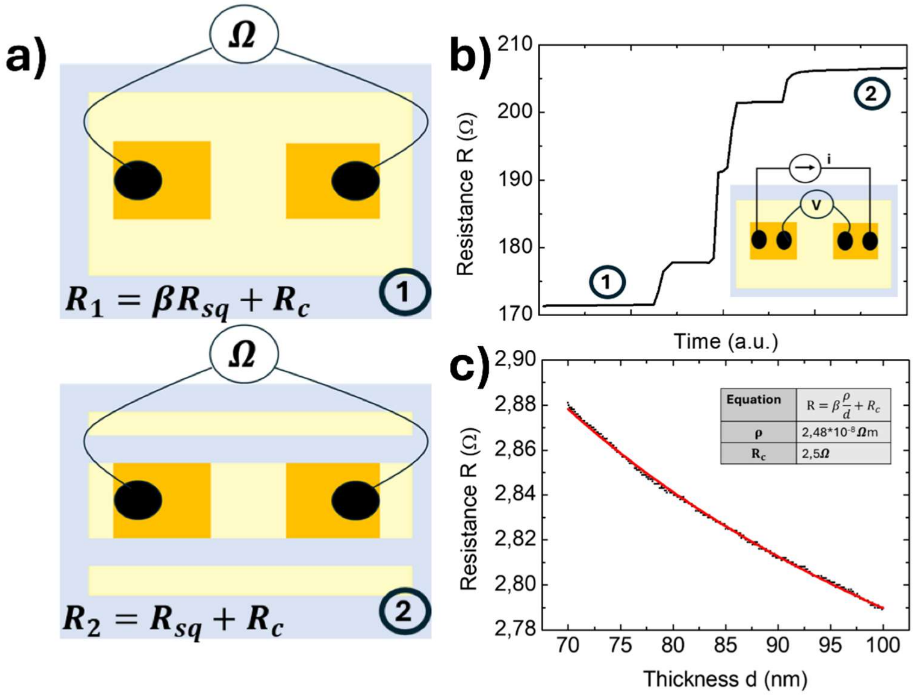

Figure S5. a) Sketch of the two-terminal configuration employed for the in-situ electrical measurements. Configuration 1 illustrates the setup used for the in-situ resistance measurement of the IBI-processed gold film, which has a non-square geometry (shown as a light yellow rectangle). Configuration 2 schematized the sample square geometry obtained ex-situ, after performing two mechanical scratches, for evaluating the geometrical factor β. b) Real time measurements of the resistance during the mechanical scratching from configuration 1 to configuration 2. Inset: contact pads employed in the in-situ IBI measurement modified for ex-situ 4-terminal resistance measurement. c) Fit of the resistance *R* vs. thickness *d,* used to determine the value of the contact resistance (R_c) and resistivity (ρ).

6. Thickness measurement after the IBI treatment of Figure 3a

In **Figure S6a**, the mechanical scratch and its corresponding height histogram (**Figure S6b**) are presented for the film obtained after the IBI treatment presented in the main text. The electrical behavior corresponding to this treatment is discussed in **Figure 3a**.

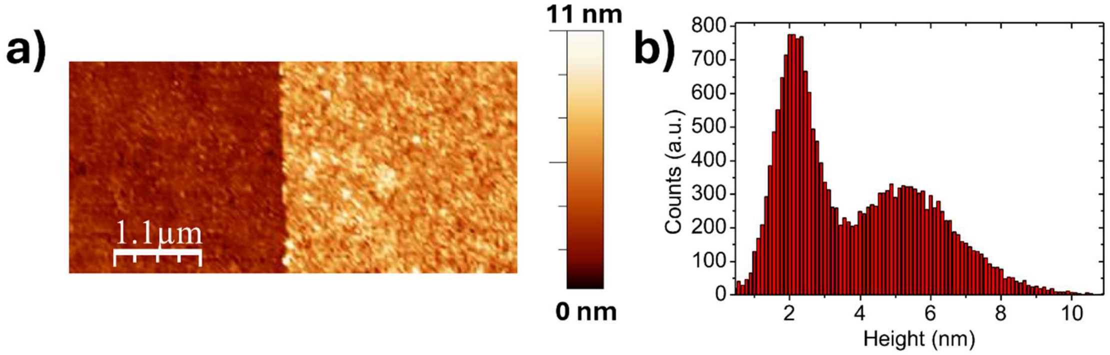

Figure S6. a) Mechanical scratch of the gold film after the IBI treatment described in Figure 3a. b) Histogram of the heights relative to the AFM image reported in panel a.

7. Sheet resistance evolution and endurance under cyclic bending

To better analyze the mechanical stability of our IBI thinned gold sample we performed a sequence of bending cycles at two different bending radii (4 mm and 6 mm) and compared the results of the resistance measurements with an ITO film (6 mm bending radius). All samples were grown on a polycarbonate substrate. In both cases, the ultra-thin gold film exhibited superior performance compared to the ITO film. Specifically, as reported in **Figure S7**, the ultra-thin gold film displayed an R/R_0 value (the ratio between resistance during bending (R)

and initial resistance (R_0)) that, after 1200 cycles, was about 6 times lower at a 4 mm radius, and about 20 times lower at a 6 mm radius compared to the behavior of the ITO film.

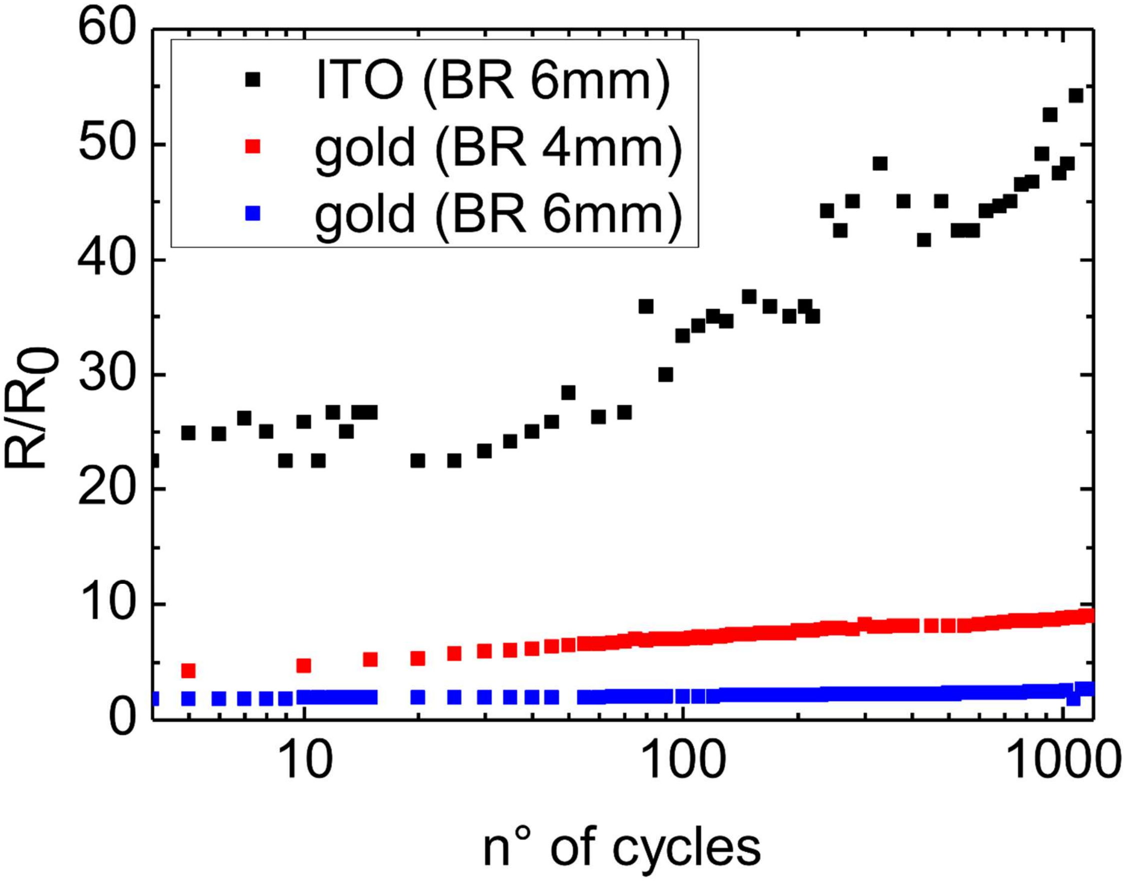

Figure S7. Comparison of film resistance (R), normalized to its initial resistance (R_0), as a function of increasing number of bending cycles. The red dots represent an ultra-thin gold film with a bending radius (BR) of 4 mm, the blue dots correspond to an ultra-thin gold film with a BR of 6 mm, and the black dots represent an ITO film with a BR of 6 mm.
